# Functional analyses of bacterial genomes found in Symbiodiniaceae genome assemblies

**DOI:** 10.1111/1758-2229.13238

**Published:** 2024-03-05

**Authors:** Eiichi Shoguchi, Masanobu Kawachi, Chuya Shinzato, Girish Beedessee

**Affiliations:** ^1^ Marine Genomics Unit, Okinawa Institute of Science and Technology Graduate University Onna Japan; ^2^ Center for Environmental Biology and Ecosystem Studies National Institute for Environmental Studies Tsukuba Japan; ^3^ Atmosphere and Ocean Research Institute, The University of Tokyo Kashiwa Japan; ^4^ Department of Biochemistry University of Cambridge Cambridge UK; ^5^ Present address: Faculty of Health & Life Sciences Northumbria University Newcastle upon Tyne UK

## Abstract

Bacterial–algal interactions strongly influence marine ecosystems. Bacterial communities in cultured dinoflagellates of the family Symbiodiniaceae have been characterized by metagenomics. However, little is known about whole‐genome analysis of marine bacteria associated with these dinoflagellates. We performed in silico analysis of four bacterial genomes from cultures of four dinoflagellates of the genera *Symbiodinium*, *Breviolum*, *Cladocopium* and *Durusdinium*. Comparative analysis showed that the former three contain the alphaproteobacterial family Parvibaculaceae and that the *Durusdinium* culture includes the family Sphingomonadaceae. There were no large genomic reductions in the alphaproteobacteria with genome sizes of 2.9–3.9 Mb, implying they are not obligate intracellular bacteria. Genomic annotations of three Parvibaculaceae detected the gene for diacetylchitobiose deacetylase (Dac), which may be involved in the degradation of dinoflagellate cell surfaces. They also had metabolic genes for dissimilatory nitrate reduction to ammonium (DNRA) in the nitrogen (N) cycle and cobalamin (vitamin B_12_) biosynthetic genes in the salvage pathway. Those three characters were not found in the Sphingomonadaceae genome. Predicted biosynthetic gene clusters for secondary metabolites indicated that the Parvibaculaceae likely produce the same secondary metabolites. Our study suggests that the Parvibaculaceae is a major resident of Symbiodiniaceae cultures with antibiotics.

## Graphical abstract

The alphaproteobacterial family Parvibaculaceae is likely a major resident of the dinoflagellate Symbiodiniaceae cultures with antibiotics. Whole‐genome analyses of the three Parvibaculaceae clarified potential molecular interaction in antibiotic‐containing media. Characteristic genes for diacetylchitobiose deacetylase (Dac), dissimilatory nitrate reduction to ammonium (DNRA) and vitamin B_12_ biosynthesis may be involved in the bacterial–algal interactions.

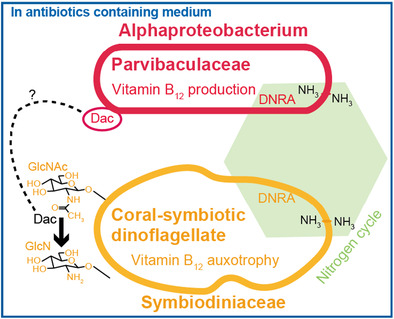

## INTRODUCTION

In aquatic ecosystems, algae co‐occur with bacterial communities (Cole, 1982). This association has evolved complex networks of interactions, mediated by diverse molecules and recognition mechanisms (Seymour et al., 2017). Algal–bacterial interactions comprise nutrient exchange, signal transduction and gene transfer (Kouzuma & Watanabe, 2015). Amin et al. (2015) clarified molecular interactions between marine algae and bacteria by using growth assays and metabolomics. The dinoflagellate family Symbiodiniaceae (symbiotic algae), intracellular residents in corals, is no exception to these patterns (Nitschke et al., 2020). Bacteria associated with Symbiodiniaceae also support coral persistence via the exchange of metabolites and bioactive compounds (Matthews et al., 2020; Silveira et al., 2017). The relationship of these bacteria with Symbiodiniaceae in acquiring, exchanging and competing for resources remains poorly understood, limiting our understanding of how microbes jointly regulate the health and viability of coral holobionts (Bernasconi et al., 2018; Matthews et al., 2020; Silveira et al., 2017). Abundant bacterial taxa from cultures of symbiotic Symbiodiniaceae have been reported (Lawson et al., 2018), and even intracellular bacteria have been observed in cultured Symbiodiniaceae (Maire et al., 2021). Identified bacteria included alphaproteobacteria, gammaproteobacteria and flavobacteria (Lawson et al., 2018; Maire et al., 2021; Shoguchi et al., 2013). Genome analyses from the Symbiodiniaceae *Breviolum* cultures in an antibiotic‐containing medium have reported sequences from an alphaproteobacterial species in addition to the *Breviolum* genome sequences (Shoguchi et al., 2013). The bacterium seems to be resistant to antibiotics. Some antibiotics and antibiotic‐producing bacteria have been detected in coral reefs (Burkholder, 1973; Zhang et al., 2018). In addition, pathogenic bacteria (such as *Vibrio*) with the different Symbiodiniaceae genera have also been detected in response studies of coral‐associated bacteria communities to heat stress and coral diseases (Littman et al., 2010; Rouzé et al., 2016). Bacterial genomes from Symbiodiniaceae cultures treated with antibiotics may provide simple useful resources to analyse potential relationships between marine bacteria and Symbiodiniaceae. To obtain the research basis for analysing bacterial–algal interactions using the simple culturing system at the molecular level, here, we annotated and examined four genomes of bacteria in Symbiodiniaceae cultures by focusing on metabolic genes and found genomic evidence supporting possible metabolic interactions between bacteria and Symbiodiniaceae.

## EXPERIMENTAL PROCEDURES

### Culturing of bacteria found in Symbiodiniaceae strains

**FIGURE 1:**
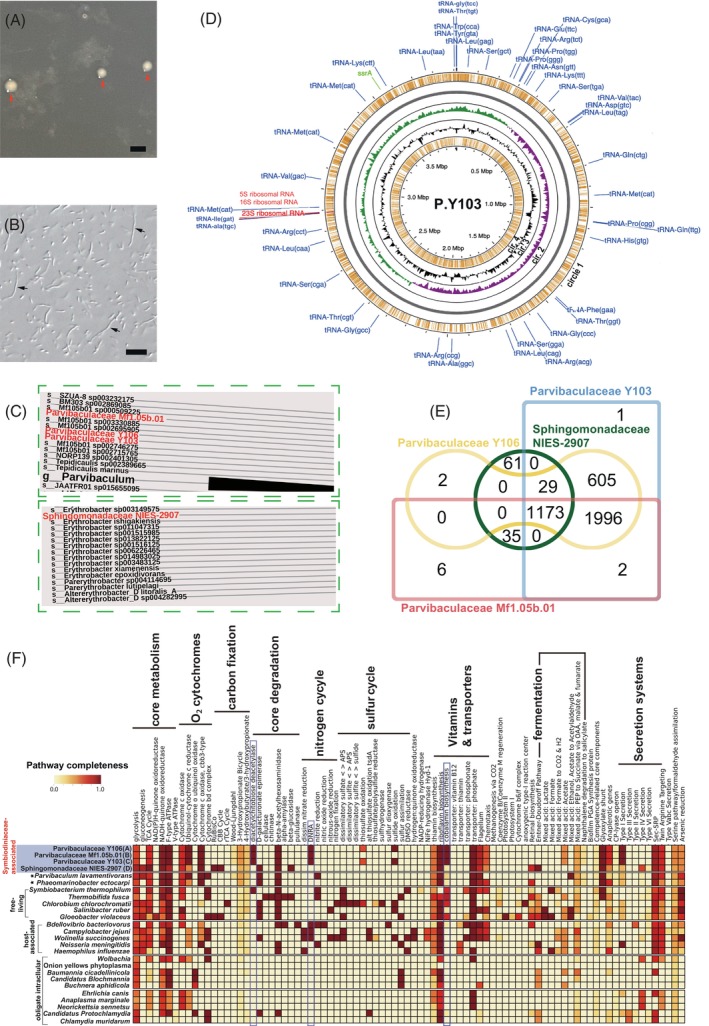
Genomic characterization of dinoflagellate‐associated bacteria. (A) White colonies with red arrows indicate isolated alphaproteobacterial Parvibaculaceae in *Cladocopium* (Symbiodiniaceae) on an agar plate. Scale bar: 1 mm. (B) Live bacterial cells from a white colony were confirmed and photographed under differential interference contrast (DIC) microscopy using a Zeiss AxioImager Z1 microscope equipped with an AxioCam digital camera (Zeiss, Jena, Germany). Cell lengths were less than 10 μm, and cell images with arrowheads are likely to be from multiple cells. Scale bar: 10 μm. (C) The classification of analysed genomes with the Genome Taxonomy Database. A part of the molecular phylogenetic tree is shown (see Figure S4 for the entire phylogenetic tree). (D) Circular plots of the 3.9‐Mb alphaproteobacterial Parvibaculaceae Y103 genome. The outermost circle (orange circle (1) shows predicted coding sequences on the forward strand. Green and purple in circle 2 indicate plus and minus GC skews, respectively. The black waveform circle 3 shows GC content. The innermost circle (orange circle 4) shows predicted coding sequences on the reverse strand. tRNA, tmRNA (transfer‐messenger RNA) and rRNA genes are shown in blue, green and red letters, respectively. (E) Venn diagrams of gene content of analysed alphaproteobacterial genomes. (F) Inferred metabolism from decoded alphaproteobacterial genomes (blue‐highlighted). Potential metabolites of four Symbiodiniaceae‐associated bacteria were predicted using Prokka annotations. Annotations for 22 other genomes are shown for comparison (Tables 1 and S1). Three metabolic pathways surrounded with blue lines are found in three Parvibaculaceae genomes, but not that of Sphingomonadaceae.

Three Symbiodiniaceae strains, *Symbiodinium tridacnidorum* Y106, *Breviolum minutum* Mf1.05b.01 and *Cladocopium* sp. Y103, were grown and maintained in an f/2 medium with three antibiotics (100 μg/mL ampicillin, 50 μg/mL streptomycin and 50 μg/mL kanamycin) (Shoguchi et al., 2018). The antibiotics have been used for obtaining Symbiodiniaceae genomes and transcriptomes because the growth of potential pathogenic bacteria with apparent harm has been avoided by using them (Bayer et al., 2012; Soffer et al., 2008). It may be possible to study the simple relationship between Symbiodiniaceae and the bacteria (Takagi et al., 2023). *Durusdinium trenchii* NIES‐2907 was cultured in f/2 medium and soil extract without antibiotics (Shoguchi et al., 2021) because the *D. trenchii* strain could not be grown in f/2 medium with antibiotics. Therefore, it is considered that there is a bias in conducting the comparative analysis with bacteria from the *Durusdinium* cultures. All cultures were maintained at 25°C on a 12‐h light/12‐h dark cycle at about 20 μmol m^−2^ s ^−1^ illumination with white fluorescent lamps in a plant growth chamber (CLE‐305, TOMY). The strain NIES‐2907 is available at the National Institute for Environmental Studies (NIES) in Tsukuba (https://mcc.nies.go.jp). In the same way, other Symbiodiniaceae strains studied here, Mf1.05b.01, Y106 and Y103, are also available as NIES‐3808, NIES‐4076 and NIES‐4077, respectively, at NIES. By simple dilution method, isolations of antibiotic‐resistant bacteria in cultures of Mf1.05b.01, Y106and Y103 were performed using 1.5% agar plates including 0.3% peptone, 0.01% yeast extract, f/2, 100 μg/mL ampicillin, 50 μg/mL streptomycin and 50 μg/mL kanamycin. An agar plate that was streaked with culture medium of *Cladocopium* sp. Y103 had white colonies, one of which was isolated and grown (Figure 1A,B). 16S rRNA genes were amplified using primers (27F: AGAGTTTGATCMTGGCTCAG and 519R: GWATTACCGC GGCKGCTG) and sequenced by the Sanger method (Lawson et al., 2018). The sequence corresponded to the genomic sequence of Parvibaculaceae Y103 and the 16S rRNA sequence of an uncultured bacterium clone, Symbiodinium_clade_C_core_32 (ID: MF598533.1) in the GenBank database (Figure S1) (Lawson et al., 2018). No other Parvibaculaceae were isolated from the culture medium of other Symbiodiniaceae strains.

### Genome annotation and metabolic potential analysis

Whole‐genome sequences from each culture indicated that the four Symbiodiniaceae cultures include bacteria. Methods for genome assembly have been described in our previous papers for draft genomes of Symbiodiniaceae strains (Shoguchi et al., 2013, 2018, 2021) because we sequenced and assembled Symbiodiniaceae and bacterial genomes at the same time. Bacterial sequences from *B. minutum* Mf1.05b.01 have been published with accession nos. BAOK01000001 and BAOK01000002 in DDBJ/EMBL/GenBank (Shoguchi et al., 2013). The sequencing technology and assembly programs were 454 GS FLX and GS De Novo Assembler v2.3 (Newbler, Roche), respectively. In this study, two sequences were linked into a single scaffold sequence for comparative analysis. Sequence technologies for *S. tridacnidorum* Y106 and *Cladocopium* sp. Y103 were Illumina GAIIx and Illumina HiSeq (Shoguchi et al., 2018). The assembly program for them was IDBA_UD (ver. 1.1.0). The sequencing technology for *D. trenchii* NIES‐2907 was Illumina HiSeq. The assembly programs were Platanus v. 1.2.4 and Newbler v. 2.9 (Shoguchi et al., 2021). Bacterial sequences from the latter three Symbiodiniaceae strains have not been published previously. Nearly complete genome sequences with 16S rRNA gene sequences were selected for in silico analysis.

For taxonomic classification with bacterial genome sequences, GTDB‐Tk (Genome Taxonomy Database Toolkit) (v2.1.0) and its latest reference database were used (Chaumeil et al., 2019). Briefly, the classify‐wf module of GTDB‐Tk was used where marker genes were identified in the four bacterial genes to generate multiple sequence alignment for determining their classification. The tree was visualized using the interactive tree of life (iTOL v6) webserver (Letunic & Bork, 2007).

All four bacterial genomes were annotated using Prokka 1.14.6 (Seemann, 2014) with NCBI compliance enabled. The completeness of assembled bacterial genomes was evaluated using CheckM v1.1.3 (Parks et al., 2015), and genomes were visualized using the GCView server (Bernasconi et al., 2018). Protein‐coding sequences were annotated against the KEGG database using KofamScan (Lawson et al., 2018) with output in mapper format, and outputs were visualized with KEGG‐Decoder (Maire et al., 2021). For comparison with bacterial genomes in Symbiodiniaceae cultures, two genomes from Parvibaculum and Phaeomarinobacter, which are other members of the same family, were selected (Dittami et al., 2014; Schleheck et al., 2011). Genomes of other 20 bacteria from different environments (Table S1), representing free‐living and host‐dependent lifestyles, were selected by referring to Merhej et al. (2009). To determine the metabolic potential of bacterial genomes, Prokka internal annotations were passed to the ModelSEED pipeline (Henry et al., 2010) using the gapfill model and a minimum reaction flux of 0.1. Potential biosynthetic gene clusters (BGCs) were identified using antiSMASH version 6 (Blin et al., 2021) with all options enabled.

### Scanning of carbohydrate‐active enzymes, ABC transporters and secretion systems

The four genomes were screened for carbohydrate‐active enzymes (CAzymes) responsible for the degradation, synthesis and modification of all carbohydrates, by searching against annotated hidden Markov model (HMM) profiles of CAZyme proteins (dbCAN HMMs v10.0) (Yin et al., 2012) using hmmscan at a cutoff *e*‐value of <10^−5^ and coverage >0.35. Potential ABC transporters were first searched for transmembrane helices within bacterial protein sequences using TMHMM v2.0 (Krogh et al., 2001). Positive sequences were then classified as transporters by performing BLASTP with an *e*‐value cutoff of 10^−5^ against Transporter Classification Database (TCDB) sequences (Saier Jr et al., 2014). By the BlastKOALA tool, secretion systems were screened using whole proteomes as queries against the taxonomic group “Prokaryotes” and “family_eukaryotes + genus_prokaryotes” in the KEGG database (Kanehisa et al., 2016). Five effector substrates datasets (T1SE‐T4SE and T6SE) obtained from BastionHub (Wang et al., 2021) were used to create HMM profiles and queried for bacterial effector proteins.

## RESULTS AND DISCUSSION

### Characterization of nearly complete genomes in bacteria cultured with Symbiodiniaceae

Bacterial genome assemblies from three cultures of Symbiodiniaceae *Symbiodinium* (culture ID: Y106), *Breviolum* (Mf1.05b.01) and *Cladocopium* (Y103) showed 100% completeness, whereas that from *Durusdinium* (NIES‐2907) was 99.09% complete (Figure S2). 16S rRNA analyses with the SILVA database (Quast et al., 2013) showed that three of these bacteria belong to the alphaproteobacterial family Parvibaculaceae, whereas the bacterium associated with *Durusdinium trenchii* likely belongs to the family Sphingomonadaceae (Table S2). These bacteria were named Parvibaculaceae Y106 (P.Y106), Parvibaculaceae Mf1.05b.01 (P.Mf1.05b.01), Parvibaculaceae Y103 (P.Y103) and Sphingomonadaceae NIES‐2907 (S.NIES‐2907), based on the Symbiodiniaceae culture ID, after the alphaproteobacterial family name. The GTDB‐Tk (Genome Taxonomy Database Toolkit) has been used to classify thousands of draft genomes (Chaumeil et al., 2019). By using GTDB‐Tk, we reconfirmed the classification of our assembled genomes. The phylogenomic tree supported that analysed genomes belong to alphaproteobacteria (Figures 1C and S3).

GC contents of the three Parvibaculaceae genomes ranged between 55.4 and 56.9%, lower than those of the alphaproteobacterial Sphingomonadaceae (58.1%) and two other Parvibaculaceae (*Phaeomarinobacter ectocarpi* and *Parvibaculum lavamentivorans*) (Table 1, Figures 1D and S4A–C). Genome annotations indicated that genomes of the Parvibaculaceae have 3700–3800 protein‐coding genes, larger than in free‐living *Parvibaculum lavamentivorans*, despite similar genome sizes. We isolated a P.Y103 clone from a *Cladocopium* culture (Figures 1A.B and S1). This culture will be a useful resource to study biotic interactions because Symbiodiniaceae‐associated Parvibaculaceae have not been isolated (Maire et al., 2021).

**TABLE 1:** Genome statistics of analysed alphaproteobacteria with two reported genomes.

	Parvibaculaceae Y106	Parvibaculaceae Mf 1.05b.01^a^	Parvibaculaceae Y103	Sphingomonadaceae NIES‐2907	*Phaeomarinobacter ectocarpi* ^b^	*Parvibaculum lavamentivorans* ^c^
Possible host strain name	*Symbiodinium tridacnidorum* Y106	*Breviolum minutum* Mf 1.05b.01	*Cladocopium* sp. Y103	*Durusdinium trenchii*	Brown alga *Ectocarpus*	Not found
Genome size (bp)	3,919,231	3,824,276	3,913,393	2,905,589	3,415,905	3,914,745
G+C mol.	56.9	55.4	56.9	58.1	58.9	62.3
Number of CDS	3827	3717	3840	2816	3259	3695
Number of genes	3874	3763	3887	2862	3304	3746
Number of rRNA	3	3	3	3	3	3
Number of tRNA	43	42	43	42	41	47
Number of tmRNA	1	1	1	1	1	1

^a^ Shoguchi et al. (2013).

^b^ Dittami et al. (2014).

^c^ Schleheck et al. (2011).

Comparative genomic characterization showed that P.Y106 and P.Y103 are conserved, except for five genes, implying they are conspecific, while P.Y103 and P.Mf1.05b.01 differ by 676 genes (~17%) (Figure 1E). Metabolic analysis indicated that P.Y106 and P.Y103 share genes for sulfur oxidation, a process that is absent in P. Mf1.05b.01 (Figure 1F). Only the three Parvibaculaceae genomes share genes for diacetylchitobiose deacetylase (Dac), DNRA (dissimilatory nitrate reduction to ammonium) and cobalamin (vitamin B_12_) biosynthesis (Figure 1F). Dac has catalytic activity for N‐acetylglucosamine (GlcNA), a sugar present on Symbiodiniaceae cell surfaces (Tortorelli et al., 2021), suggesting a role of Dac in interactions between Symbiodiniaceae and Parvibaculaceae. Partial degradation of Symbiodiniaceae cell surfaces by bacterial Dac may enable recognition of Symbiodiniaceae by corals (Takeuchi et al., 2021), implying the possibility that these are epibiotic bacteria. DNRA activity is likely important for coral reef nitrogen cycling (Glaze et al., 2022) and in Parvibaculaceae–Symbiodiniaceae relationships because they have been detected in genomes of other host‐associated bacteria (*Bdellovibrio*, *Campylobacter* and *Wolinella*) (Figure 1F). Furthermore, metabolic network analysis revealed possible differences in the nitrogen cycle between Parvibaculaceae (Figure S5A) and Sphingomonadaceae (Figure S5B), in which the metabolic network in the Parvibaculaceae functions in NH_3_ transport (Figure S5A). On the other hand, the genome of Sphingomonadaceae NIES‐2907 likely encodes genes for Rubisco and the Calvin–Benson–Bassham (CBB) cycle in carbon fixation (Figure 1F). Interestingly, similarities to globally distributed bacterial phototrophs suggest a possible new source of photosynthesis in the ocean (Graham et al., 2018).

**FIGURE 2:**
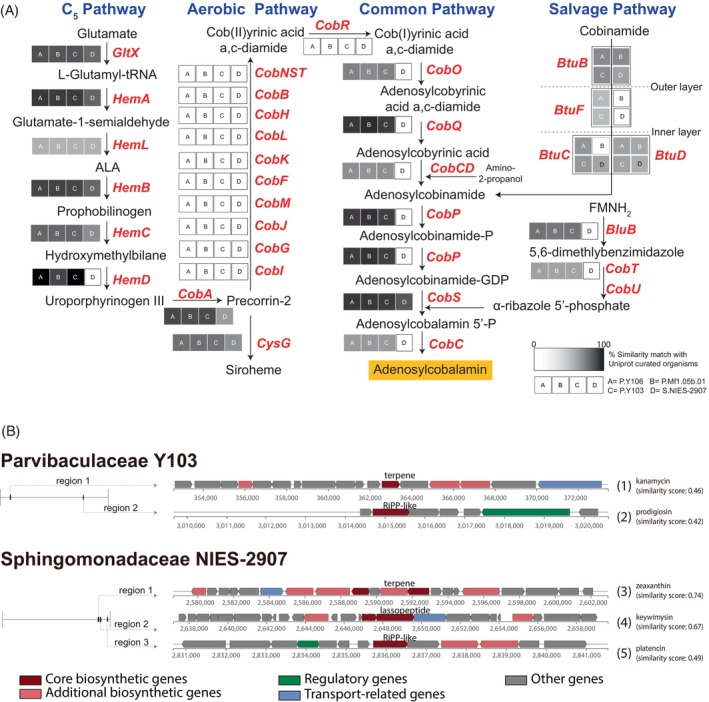
Putative biosynthetic genes for secondary metabolites from genomes of major dinoflagellate‐associated bacteria. (A) Biosynthetic pathways of vitamin B_12_ and putative enzymatic genes show that salvage pathways biosynthesize adenosylcobalamin in Parvibaculaceae P.Y106 and P.Y103. (B) Putative biosynthetic gene clusters in genomes of Symbiodiniaceae‐associated bacteria (P.Y103 and S.NIES‐2907). More than 80% of genes in clusters, except the lasso peptide biosynthetic gene cluster (region 2 in NIES‐2907), showed similarities to clustered genes in known bacterial genomes (see Figure S6). Predicted links between gene clusters and secondary metabolites are shown by the numbers in parentheses.

Vitamin B_12_‐auxotrophy has been reported in dinoflagellates (Lin et al., 2022). Therefore, vitamin B_12_ from Parvibaculaceae is likely used by dinoflagellates. In a detailed analysis, we found that genes involved in the B_12_ aerobic or anaerobic pathway were absent, whereas several genes involved in salvage and remodelling processes were identified, suggesting that Parvibaculaceae species salvage intermediate cobinamide and employ them in cobalamin biosynthesis (Figure 2A). On the other hand, the genome of Sphingomonadaceae NIES‐2907 does not encode genes for vitamin B_12_ biosynthesis.

Functional annotation by antiSMASH revealed the presence of biosynthetic clusters belonging to RiPP‐like (ribosomally synthesized and post‐translationally modified peptide‐like) and terpene biosynthesis (Figures 2B and S6A). Gene clusters had similarities with those of other alphaproteobacteria (Figure S6B). Prodigiosin is likely produced by many bacterial species, including the three Parvibaculaceae, and is known to impregnate cellulose matrices, acting as a potent antimicrobial agent (Danevčič et al., 2016). A closely related member, cycloprodigiosin, reportedly has a positive settling effect on larvae of the coral, *Leptastrea purpurea* (Petersen et al., 2021). In contrast, the Sphingomonadaceae contain candidate gene clusters for possible zeaxanthin production, which is thought to alleviate thermal and light stress in Symbiodiniaceae (Motone et al., 2020; Takagi et al., 2023).

### Additional candidate genes related to host association in four bacterial genomes

Genome‐wide surveys of the four genomes for CAZymes recovered multiple functional classes, predominantly glycoside hydrolases (GH), glycosyltransferases (GTs), carbohydrate esterases (CEs) and auxiliary activities (AAs), with the minor representation of carbohydrate‐binding modules (CBMs) and polysaccharide lyases (PLs) (Table S3). This suggests the ability of Parvibaculaceae and Sphingomonadaceae bacteria to metabolize different types of carbohydrates in diverse habitats. Among the Parvibaculaceae, glycosyltransferase family 2, members of which catalyse the formation of glycosidic linkages, was the most abundant shared GT family, suggesting a role in the Parvibaculaceae–Symbiodiniaceae association. In contrast, in the Sphingomonadaceae, CE Family 1, enzymes that release acyl or alkyl groups attached by ester linkage to carbohydrates, were more abundant.

Eukaryotic‐like protein domains such as ANKs, TPRs, Sel1‐like repeats, Fn3‐like and bacterial Ig‐like domains involved in protein–protein interactions were detected in almost all genomes analysed (Table S4). An abundance of such proteins has been found in many sponge‐associated microbes, implying the role of proteins containing such domains for successful host association (Fan et al., 2012; Liu et al., 2011). In addition, we also found TadE‐like domains, responsible for surface adherence and binding. Most abundant were members of the LuxR protein family, which serve regulatory functions in quorum sensing to regulate metabolite secretion (Duerkop et al., 2009). Overall, the domains identified here support the ability of Parvibaculaceae and Sphingomonadaceae to attach and survive on various hosts, including dinoflagellates of the family Symbiodiniaceae.

Bacterial ABC transporters facilitate nutrient uptake, toxin secretion and quorum sensing (Davidson & Chen, 2004). We found that all four bacterial genomes possess similar transporters, with the Sphingomonadaceae exclusively having transporters for osmoprotectant and lipopolysaccharides, indicating that these bacterial taxa encounter different environmental conditions (Figure S7).

Secretion of protein or toxin‐effector molecules and their transport through the membrane via secretion systems (SS) is an important strategy for successful colonization (Merhej et al., 2009; Souza et al., 2015). Our analysis revealed that Type I SS and Type IV SS are unique to Parvibaculaceae and Sphingomonadaceae NIES‐2907, respectively (Figures 1F and S8), suggesting the difference in host interaction between Parvibaculaceae and Sphingomonadaceae.

To our knowledge, these findings provide the first genomic insights into how bacteria associated with symbiotic members of the family Symbiodiniaceae contribute to the establishment of this vital relationship with an exchange of important metabolites. One of the most abundant marine bacterial groups is the alphaproteobacteria. The sequences of Parvibaculaceae have been detected in previous reports although they were assigned as unclassified Rhodospirillaceae using SILVA (v128) (Lawson et al., 2018). Members of the family Parvibaculaceae may provide vitamin B_12_ for symbiotic dinoflagellates in marine environments (Lin et al., 2022). Further genome‐wide surveys of genes likely related to host association have been discussed on alphaproteobacterial Parvibaculaceae and alphaproteobacterial Sphingomonadaceae (Lawson et al., 2018; Maire et al., 2021). In addition, preliminary analysis of metagenomes from the *Durusdinium* culture indicated the presence of the alphaproteobacterial Parvibaculaceae (data not shown). Therefore, members of the Parvibaculaceae are likely to be common residents of Symbiodiniaceae cultures. Thus, genome annotations of Parvibaculaceae in Symbiodiniaceae cultures likely provide an initial step for studying molecular interactions between alphaproteobacteria and Symbiodiniaceae.

## AUTHOR CONTRIBUTIONS

**Eiichi Shoguchi:** Conceptualization (lead); data curation (equal); formal analysis (equal); funding acquisition (equal); writing – original draft (equal); writing – review and editing (equal). **Masanobu Kawachi:** Resources (lead); validation (equal); writing – review and editing (equal). **Chuya Shinzato:** Data curation (equal); formal analysis (equal); funding acquisition (equal); writing – review and editing (equal). **Girish Beedessee:** Data curation (equal); formal analysis (equal); validation (equal); visualization (lead); writing – original draft (equal); writing – review and editing (equal).

## CONFLICT OF INTEREST STATEMENT

The authors have no conflict of interest to declare.

## Supporting information


**Data S1.** Supporting Information.

## Data Availability

All the assembled bacterial genomes have been deposited in DDBJ, and accession numbers in DDBJ/EMBL/GenBank are LC727611 for Parvibaculaceae Y106, BAOK01000001 and BAOK01000002 for Parvibaculaceae Mf1.05b.01, LC727612 for Parvibaculaceae Y103 and LC727613 for Sphingomonadaceae NIES‐2907. Other data analysed during this study are indicated in this published article and the Supporting information.
