# Supplementary material for: Functional analyses of bacterial genomes found in Symbiodiniaceae genome assemblies
Source: Environ Microbiol Rep. 2024 Mar 5;16(2):e13238. doi: 10.1111/1758-2229.13238 (PMC10915500; doi:10.1111/1758-2229.13238)
Supplement: Supplementary file 1 — Data S1. Supporting Information. [file EMI4-16-e13238-s001.pdf]

Supporting information

```
Query: Parvibaculaceae Y103 16S rRNA Query ID: lcl|Query_160979 Length: 425


>Uncultured bacterium clone Symbiodinium_clade_C_core_32 16S ribosomal RNA gene
partial sequence
Sequence ID: MF598533.1 Length: 483
Range 1: 47 to 471

Score:785 bits(425), Expect:0.0,
Identities:425/425(100%),  Gaps:0/425(0%), Strand: Plus/Plus

Query  1    CATGCAAGTCGAACGAGAAGCTCACTTCGGTGAGTGGAAAGTGGCAGACGGGTGAGTAAC  60
            ||||||||||||||||||||||||||||||||||||||||||||||||||||||||||||
Sbjct  47   CATGCAAGTCGAACGAGAAGCTCACTTCGGTGAGTGGAAAGTGGCAGACGGGTGAGTAAC  106

Query  61   GCGTGGGAATATACCCAGAGGTACGGAACAACGCCGGGAAACTGGTGCTAATACCGTATG  120
            ||||||||||||||||||||||||||||||||||||||||||||||||||||||||||||
Sbjct  107  GCGTGGGAATATACCCAGAGGTACGGAACAACGCCGGGAAACTGGTGCTAATACCGTATG  166

Query  121  TGCCCTTCGGGGGAAAGATTTATCGCCTTTGGATTAGCCCGCGTTAGATTAGCTTGTTGG  180
            ||||||||||||||||||||||||||||||||||||||||||||||||||||||||||||
Sbjct  167  TGCCCTTCGGGGGAAAGATTTATCGCCTTTGGATTAGCCCGCGTTAGATTAGCTTGTTGG  226

Query  181  TGAGGTAAAAGCTCACCAAGGCGACGATCTATAGCTGGTCTGAGAGGATGATCAGCCACA  240
            ||||||||||||||||||||||||||||||||||||||||||||||||||||||||||||
Sbjct  227  TGAGGTAAAAGCTCACCAAGGCGACGATCTATAGCTGGTCTGAGAGGATGATCAGCCACA  286

Query  241  CTGGGACTGAGACACGGCCCAGACTCCTACGGGAGGCAGCAGTGGGGAATCTTGCACAAT  300
            ||||||||||||||||||||||||||||||||||||||||||||||||||||||||||||
Sbjct  287  CTGGGACTGAGACACGGCCCAGACTCCTACGGGAGGCAGCAGTGGGGAATCTTGCACAAT  346

Query  301  GGGCGAAAGCCTGATGCAGCCATGCCGCGTGTGTGATGAAGGCCCTAGGGTTGTAAAACA  360
            ||||||||||||||||||||||||||||||||||||||||||||||||||||||||||||
Sbjct  347  GGGCGAAAGCCTGATGCAGCCATGCCGCGTGTGTGATGAAGGCCCTAGGGTTGTAAAACA  406

Query  361  CTTTCAGTGGGGAAGATAATGACGGTACCCACAGAAGAAGCTCCGGCTAACTCCGTGCCA  420
            ||||||||||||||||||||||||||||||||||||||||||||||||||||||||||||
Sbjct  407  CTTTCAGTGGGGAAGATAATGACGGTACCCACAGAAGAAGCTCCGGCTAACTCCGTGCCA  466

Query  421  GCCGC  425
            |||||
Sbjct  467  GCCGC  471
```

FIGURE S1 The 16S rRNA sequence of isolated alphaproteobacterial Parvibaculaceae in a *Cladocopium* sp. Y103 culture (Symbiodiniaceae). Alignment to the GeneBank sequence (ID: MF598533.1) shows that the Parvibaculaceae has been also found in other *Cladocopium* cultures.

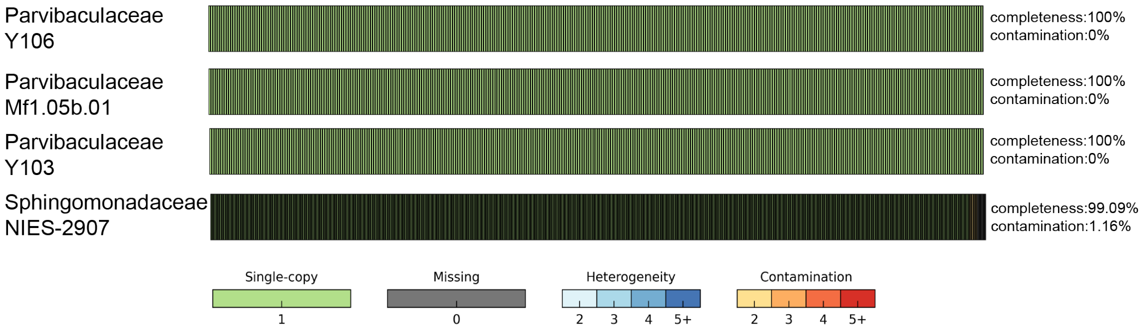

FIGURE S2 Completeness of assembled bacterial genomes. Evaluations using CheckM v1.13 indicate that Parvibaculaceae genomes are of high quality and that the Sphingomonadaceae genome is incomplete.

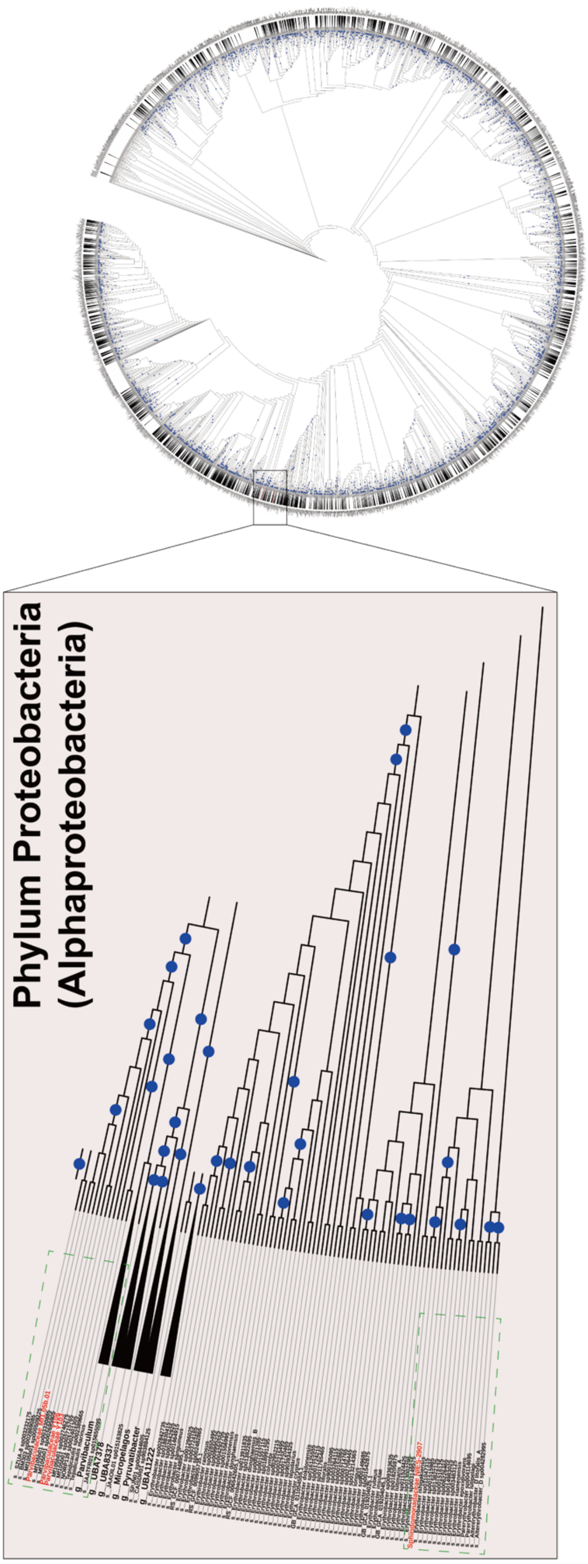

FIGURE S3 A molecular phylogenetic tree of concatenated proteins with 3,654 branches by the Genome Taxonomy Database Toolkit (GTDB-Tk release207_v2). It supported that four analyzed bacteria that are shown by red colored names belong to Alphaproteobacteria.

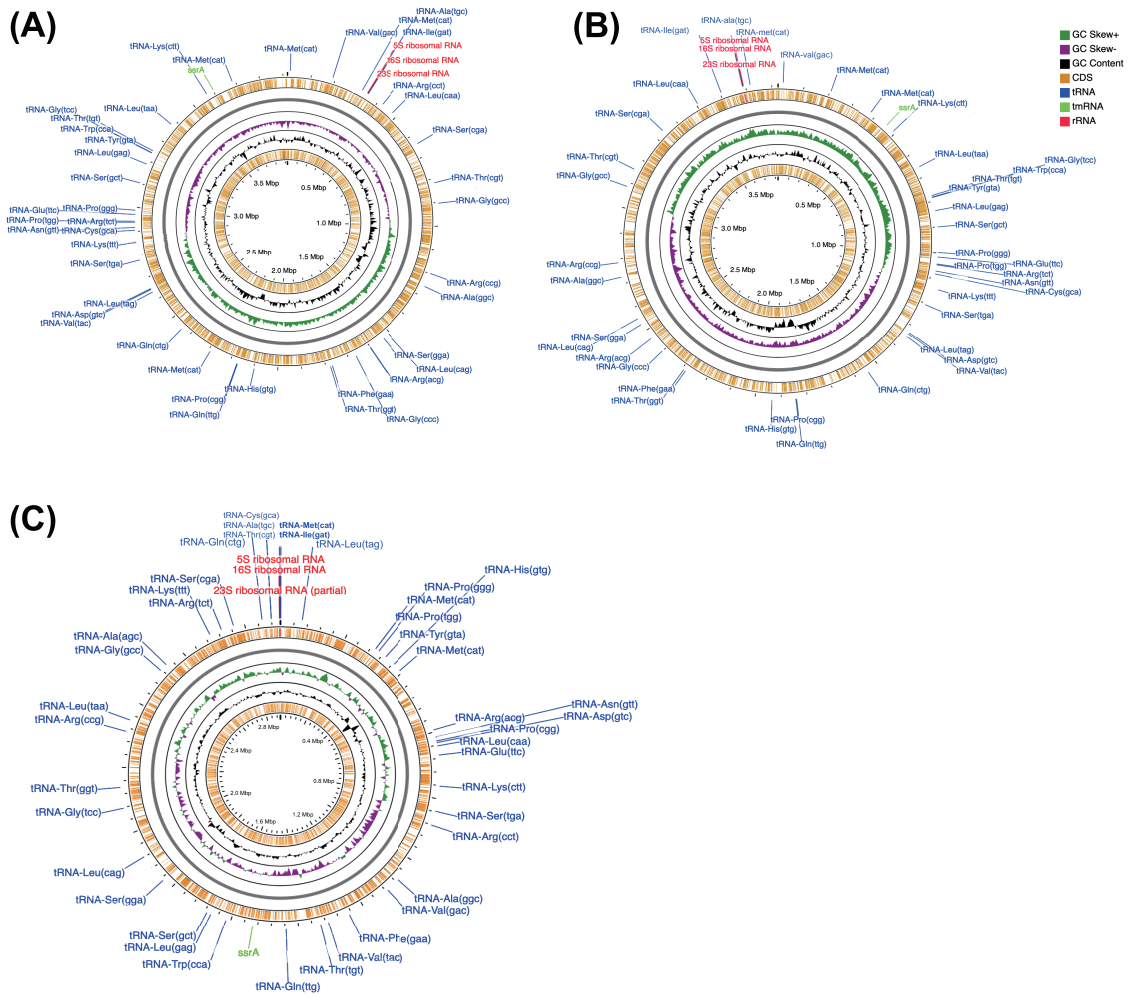

FIGURE S4 Circular maps of Symbiodiniaceae-associated alphaproteobacterial genomes. Circular genome plots of the alphaproteobacterial Parvibaculaceae Y106 (A), Parvibaculaceae Mf1.05b.01 (B), and Sphingomonadaeae NIES-2907 (C). The four circles from outermost to innermost depict gene locations or genome features. The outermost circle (orange circle 1) shows predicted coding sequences on the forward strand. Green and purple in circle 2 indicate plus and minus GC skews, respectively. The black waveform circle 3 shows GC content. The innermost circle (orange circle 4) shows predicted coding sequences on the reverse strand. The tRNA, tmRNA (transfer-messenger RNA), and rRNA genes are shown in blue, green, and red, respectively.

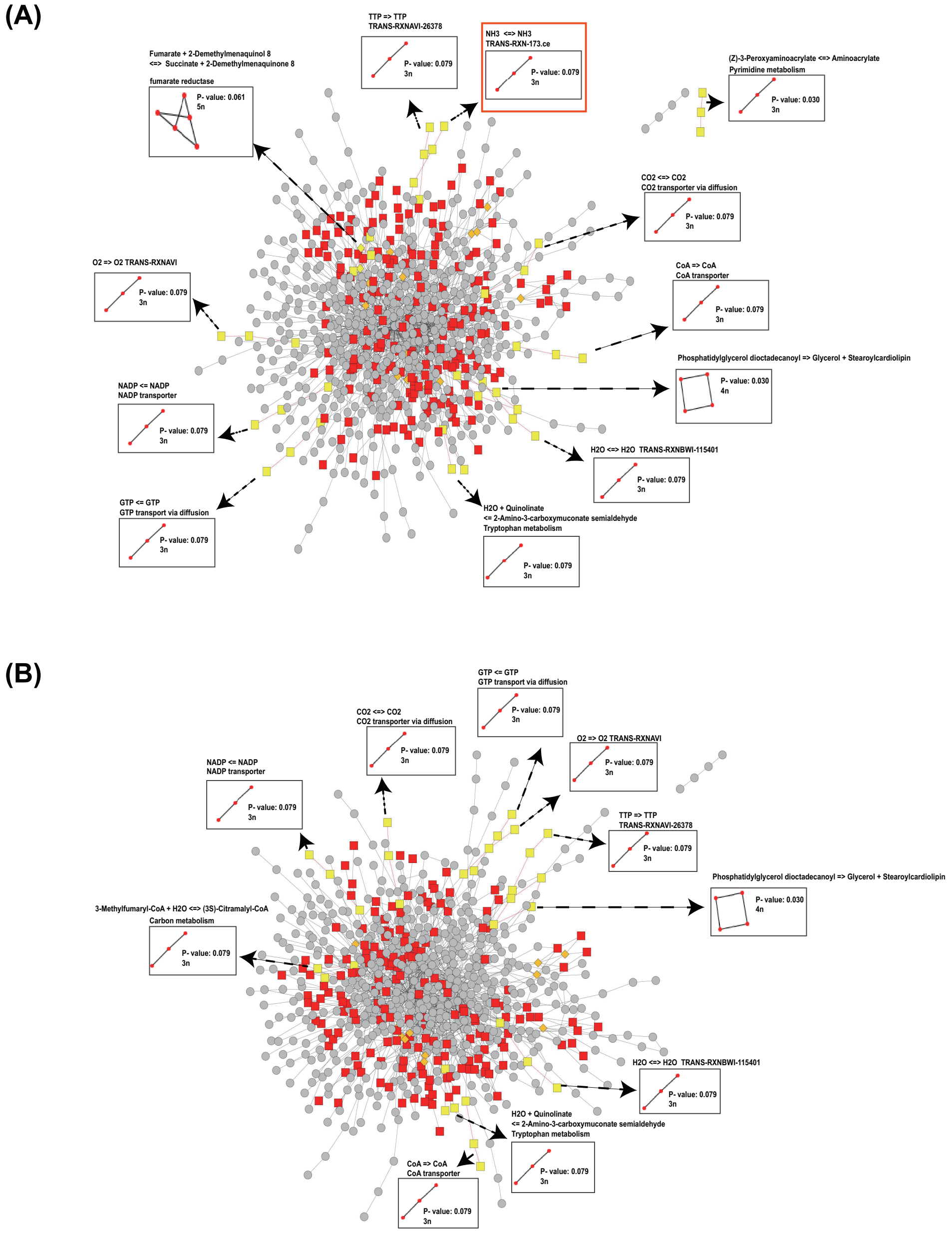

FIGURE S5 Predicted metabolic networks by the ClusterONE algorithm.

(A) Metabolic networks of Parvibaculaceae Y106 show 74 clustered (colored squares) detected with the ClusterONE algorithm. Insets in the periphery show details of clusters in yellow with p-values < 0.1 derived from the network, where n is the number of nodes. Reactions and KEGG pathways/roles of individual clusters are shown above each inset. The metabolic network for ammonia-transfer was characteristic of Parvibaculaceae genomes and is surrounded with a red line.

(B) Metabolic networks of Sphingomonadaeae NIES-2907 show 67 clusters (colored squares) detected using the ClusterONE algorithm.

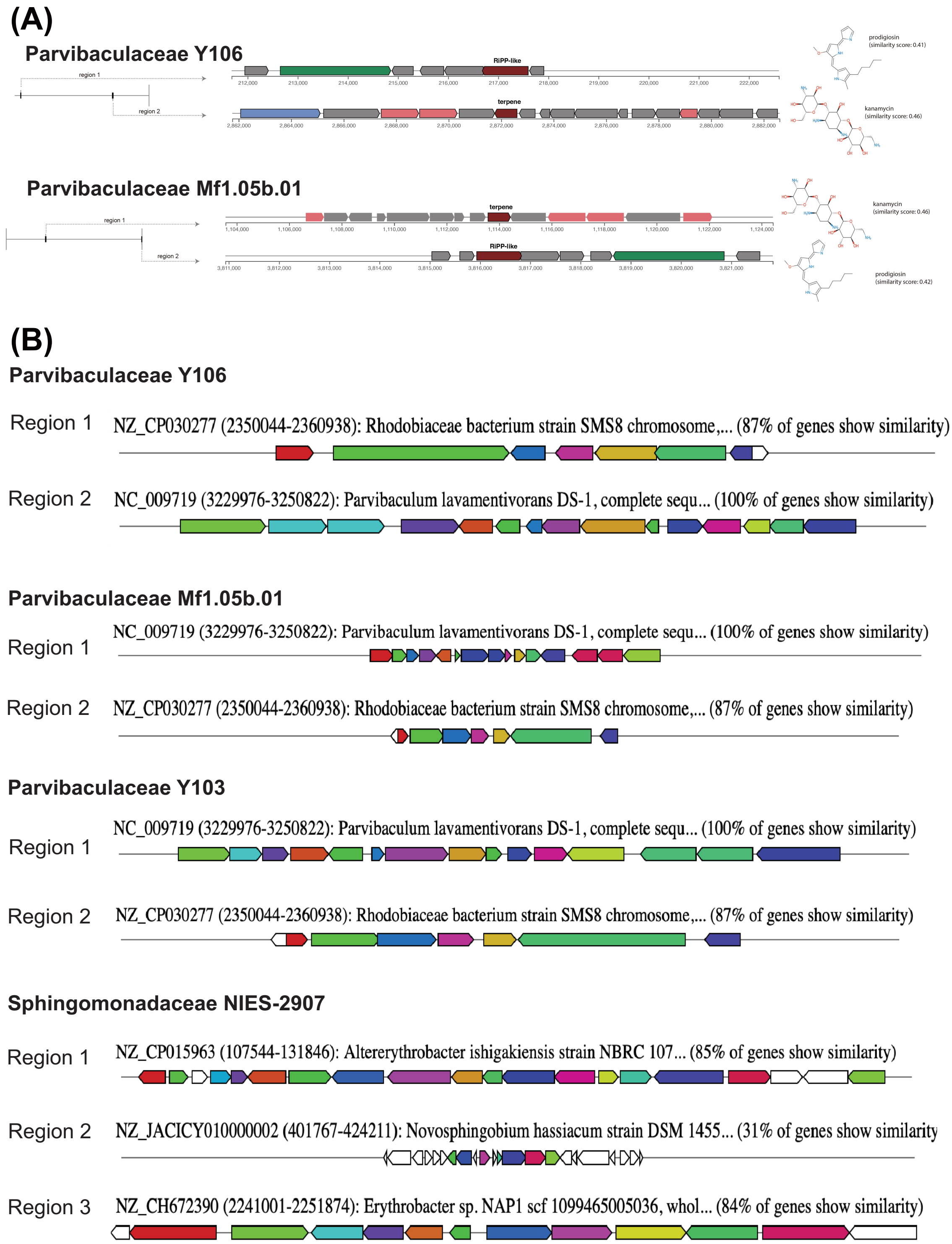

FIGURE S6 ClusterBlast alignment of gene clusters homologous to the query gene cluster from four Symbiodiniaceae-associated bacterial genomes. (A) Putative biosynthetic gene clusters in genomes of Symbiodiniaceae-associated bacteria (P.Y106 and P.Mf1.05b.01). See Figure 2B for P.Y103 and S.NIES-2907. (B) In this case, top hits belonging to secondary metabolic regions are displayed.

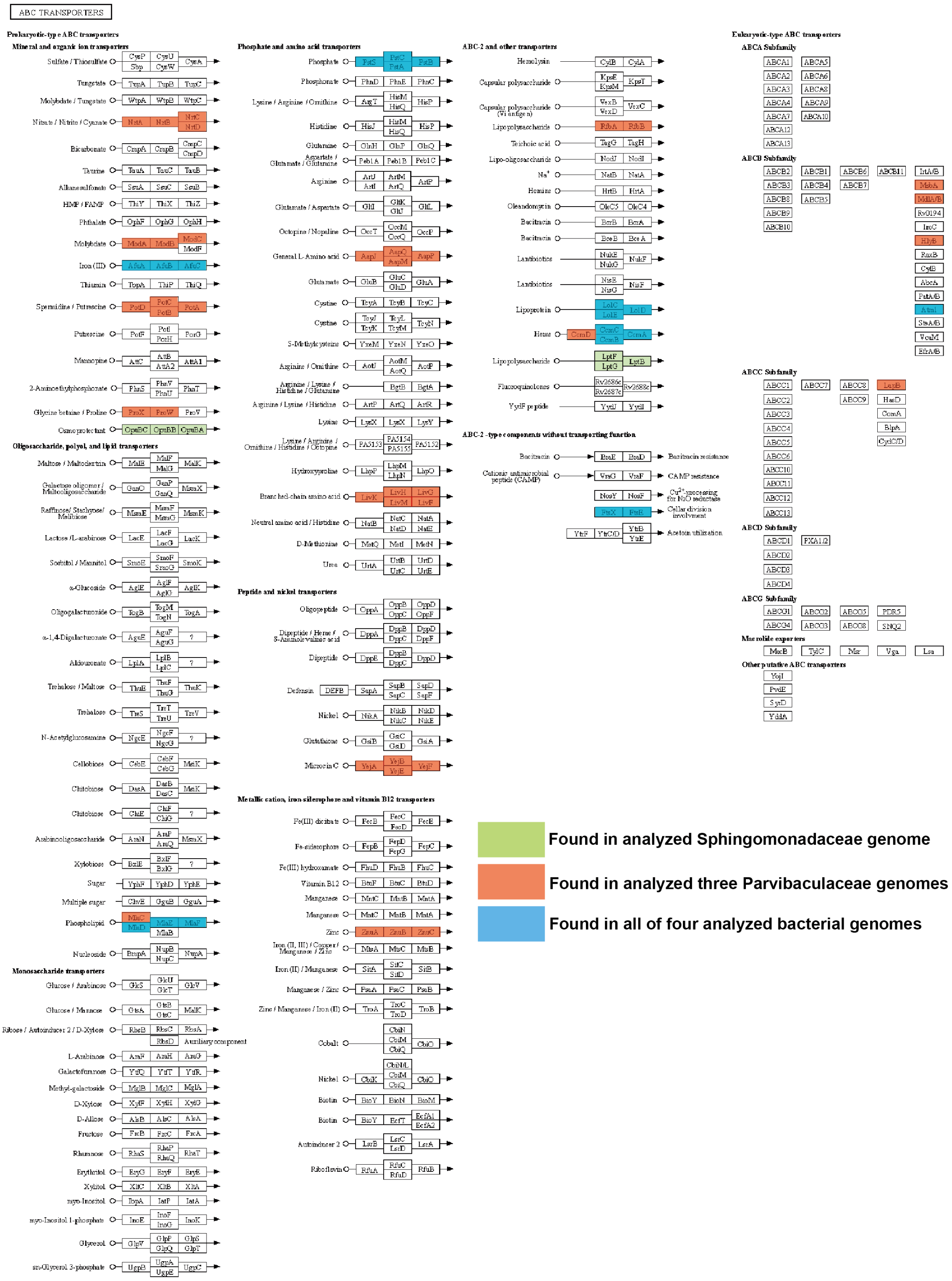

FIGURE S7 Comparison of a secretion system in the four bacterial genomes using BlastKOALA tool. Colored boxes show common, as well as specific protein groups detected in these genomes.

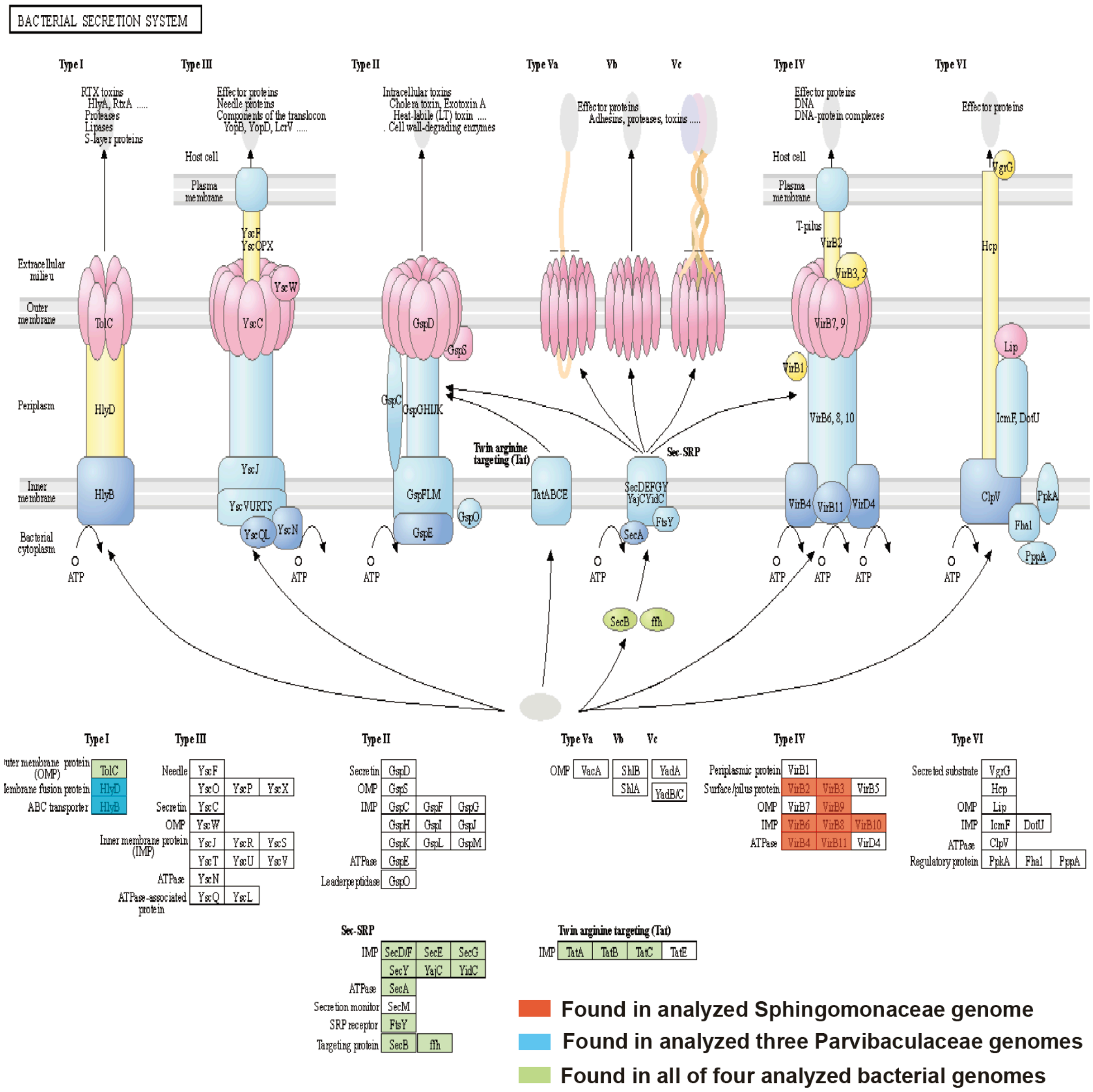

FIGURE S8 Comparison of the secretion system in four bacterial genomes using the BlastKOALA tool. Colored boxes show common as well as specific protein groups detected in these genomes.

TABLE S1 Genome statistics in bacteria shown in Figure 1F.

	Anaplasma marginale	*Baumannia cicadellinicola*	*Bdellovibrio bacteriovorus*	*Buchnera aphidicola*	*Campylobacter jejuni*	*Candidatus Blochmannia floridanus*	*Candidatus Protochlamydia amoebophila*	*Chlamydia muridarum*	*Chlorobium chlorochromatii*	*Ehrlichia canis Jake*
Genome size (bp)	1,197,687	686,194	3,782,950	645,334	1,777,831	705,557	2,438,912	1,072,986	2,572,079	1,315,030
Number of CDS	1012	606	3563	577	1871	601	1926	909	2094	970
Number of genes	1053	652	3606	613	1922	642	1973	953	2143	1011
Number of rRNA	3	6	6	3	6	3	9	6	2	3
Number of tRNA	37	39	36	32	44	37	37	37	46	37
Number of tmRNA	1	1	1	1	1	1	1	1	1	1
Accession number	NC_004842.2	CP000238.1	NC_005363.1	NZ_CP033006.1	NC_003912.7	BX248583.1	NC_005861.2	NZ_CP027211.1	NC_007514.1	NC_007354.1
References	Brayton et al. (2005)	Wu et al. (2006)	Rendulic et al. (2004)	Shigenobu et al. (2000)	Fouts et al. (2005)	Gil et al. (2003)	Horn et al. (2004)	O'Connell et al. (2006)	Méndez-Alvarez et al. (1995)	Mavromatis et al. (2006)

	Gloeobacter violaceus	*Haemophilus influenzae*	*Neisseria_meningitidis*	*Neorickettsia sennetsu*	*Onion yellows phytoplasma*	*Salinibacter ruber*	*Symbiobacterium thermophilum*	*Thermobifida fusca*	*Wolbachia*	*Wolinella succinogenes*
Genome size (bp)	4659019	1830138	2184406	859006	853092	3739924	3566135	3642249	1330657	2110355
Number of CDS	4511	1748	2052	780	954	3093	3290	3122	1304	2074
Number of genes	4562	1825	2123	816	990	3144	3412	3194	1342	2122
Number of rRNA	2	19	12	3	4	3	19	12	3	6
Number of tRNA	48	57	58	33	32	47	103	59	34	41
Number of tmRNA	1	1	1	NA	NA	1	NA	1	1	1
Accession number	NC_005125.1	L42023.1	NC_003116.1	NC_007798.1	AP006628.2	NZ_CP030356.1	NC_006177.1	NC_007333.1	NZ_CP046921.1	NC_005090.1
References	Nakamura et al. (2003)	Fleischmann et al. (1995)	Parkhill et al. (2000)	Dunning et al. (2006)	Oshima et al. (2004)	Mongodin et al. (2005)	Ueda et al. (2004)	Lykidis et al. (2007)	Wu et al. (2004)	Baar et al. (2003)

TABLE S2 Bacterial classification using the SILVA database.

	lca_tax_gtdb	lca_tax_rdp	lca_tax_slv
16S rRNA from *Symbiodinium* Y106 culture	d__Bacteria;	Bacteria;	Bacteria;
(A3_sc1_348944-347481)	p__Proteobacteria;	"Proteobacteria";	Proteobacteria;
	c__Alphaproteobacteria;	Alphaproteobacteria;	Alphaproteobacteria;
	o__Parvibaculales;	Rhizobiales;	Parvibaculales;
	f__Parvibaculaceae;	Rhodobiaceae;	Parvibaculaceae;
	g__Mf105b01;	unclassified_Rhodobiaceae;	Mf105b01;
	s__Mf105b01 sp000509225;		
16S rRNA from *Breviolum* Mf1.05b.01 culture	d__Bacteria;	Bacteria;	Bacteria;
(B1_sc1_3688383-3689848)	p__Proteobacteria;	"Proteobacteria";	Proteobacteria;
	c__Alphaproteobacteria;	Alphaproteobacteria;	Alphaproteobacteria;
	o__Parvibaculales;	Rhizobiales;	Parvibaculales;
	f__Parvibaculaceae;	Rhodobiaceae;	Parvibaculaceae;
	g__Mf105b01;	unclassified_Rhodobiaceae;	Mf105b01;
	s__Mf105b01 sp000509225;		
16S rRNA from *Cladocopium* Y103 culture	d__Bacteria;	Bacteria;	Bacteria;
(C92_sc1_2885743-2887206)	p__Proteobacteria;	"Proteobacteria";	Proteobacteria;
	c__Alphaproteobacteria;	Alphaproteobacteria;	Alphaproteobacteria;
	o__Parvibaculales;	Rhizobiales;	Parvibaculales;
	f__Parvibaculaceae;	Rhodobiaceae;	Parvibaculaceae;
	g__Mf105b01;	unclassified_Rhodobiaceae;	Mf105b01;
	s__Mf105b01 sp000509225;		
16S rRNA from *Durusdinium* NIES-2907 culture	d__Bacteria;	Bacteria;	Bacteria;
(D1_sc5_2901591-2902760)	p__Proteobacteria	"Proteobacteria";	Proteobacteria;
	c__Alphaproteobacteria	Alphaproteobacteria;	Alphaproteobacteria;
	o__Sphingomonadales	Sphingomonadales;	Sphingomonadales;
	f__Sphingomonadaceae	Erythrobacteraceae;	Sphingomonadaceae;
	g__Erythrobacter		
	s__Erythrobacter sp001542875;		

TABLE S3 Numbers of carbohydrate-active enzymes encoded in four bacterial genomes.

	Parvibaculaceae Y106	Parvibaculaceae Mf 1.05b.01	Parvibaculaceae Y103	Sphingomonadaceae NIES-2907
Glycoside hydrolases (GH)	20	24	20	17
glycosyltransferases (GTs)	31	23	31	16
Polysaccharide lyases (PLs)	3	3	3	7
Carbohydrate esterases (CEs)	25	27	25	14
Auxiliary activities (AAs)	12	15	15	6
Carbohydrate binding modules (CBMs)	3	2	3	3

TABLE S4 Numbers of predicted alphaproteobacterial proteins with domains involved in protein-protein interactions.

Protein family	domain ID	Parvibaculaceae Y106	Parvibaculaceae Mf 1.05b.01	Parvibaculaceae Y103	Sphingomonadaceae NIES-2907
tetratricopeptide repeat proteins	IPR011990	0	0	0	0
	IPR011717	1	0	1	1
	IPR001440	2	3	2	0
	IPR013105	0	2	0	1
	IPR019734	2	5	2	1
ankyrin-repeat proteins	IPR020683	1	1	1	0
	IPR002110	0	0	0	0
proteins with SEL1 repeats	IPR006597	6	6	6	0
leucine-rich repeats (LRRs)		0	0	0	0
laminin G domains		0	0	0	0
fibronectin type III (fn3) domains	IPR026891	1	0	1	0
bacterial Ig-like domains	IPR041498	2	2	2	2
proteins with the TadE-like domain	IPR012495	2	2	2	2
invasion protein B	IPR010642	1	1	1	0
LuxR	IPR000792	10	8	10	6
	IPR005143	1	0	1	1

REFERENCES

Baar, C., Eppinger, M., Raddatz, G., Simon, J., Lanz, C., Klimmek, O., et al. (2003) Complete genome sequence and analysis of Wolinella succinogenes. *Proc Natl Acad Sci U S A* 100, 11690–11695.

Brayton, K.A., Kappmeyer, L.S., Herndon, D.R., Dark, M.J., Tibbals, D.L., Palmer, G.H., et al. (2005) Complete genome sequencing of Anaplasma marginale reveals that the surface is skewed to two superfamilies of outer membrane proteins. *Proc Natl Acad Sci U S A* 102, 844–849.

Dunning Hotopp, J.C., Lin, M., Madupu, R., Crabtree, J., Angiuoli, S.V., Eisen, J.A., et al. (2006) Comparative genomics of emerging human ehrlichiosis agents. *PLoS Genet* 2, e21.

Fleischmann, R.D., Adams, M.D., White, O., Clayton, R.A., Kirkness, E.F., Kerlavage, A.R., et al. (1995) Whole-genome random sequencing and assembly of Haemophilus influenzae Rd. *Science* 269, 496–512.

Fouts, D.E., Mongodin, E.F., Mandrell, R.E., Miller, W.G., Rasko, D.A., Ravel, J., et al. (2005) Major structural differences and novel potential virulence mechanisms from the genomes of multiple campylobacter species. *PLoS Biol* 3, e15.

Gil, R., Silva, F.J., Zientz, E., Delmotte, F., González-Candelas, F., Latorre, A., et al. (2003) The genome sequence of Blochmannia floridanus: comparative analysis of reduced genomes. *Proc Natl Acad Sci U S A* 100, 9388–9393.

Horn, M., Collingro, A., Schmitz-Esser, S., Beier, C.L., Purkhold, U., Fartmann, B., et al. (2004) Illuminating the evolutionary history of chlamydiae. *Science* 304, 728–730.

Lykidis, A., Mavromatis, K., Ivanova, N., Anderson, I., Land, M., DiBartolo, G., et al. (2007) Genome sequence and analysis of the soil cellulolytic actinomycete Thermobifida fusca YX. *J Bacteriol* 189, 2477–2486.

Mavromatis, K., Doyle, C.K., Lykidis, A., Ivanova, N., Francino, M.P., Chain, P., et al. (2006) The genome of the obligately intracellular bacterium Ehrlichia canis reveals themes of complex membrane structure and immune evasion strategies. *J Bacteriol* 188, 4015–4023.

Méndez-Alvarez, S., Pavoń, V., Esteve, I., Guerrero, R., and Gaju, N. (1995) Genomic heterogeneity in Chlorobium limicola: chromosomic and plasmidic differences among strains. *FEMS Microbiol Lett* 134, 279–285.

Mongodin, E.F., Nelson, K.E., Daugherty, S., Deboy, R.T., Wister, J., Khouri, H., et al. (2005) The genome of Salinibacter ruber: convergence and gene exchange among hyperhalophilic bacteria and archaea. *Proc Natl Acad*

Sci U S A 102, 18147–18152.

Nakamura, Y., Kaneko, T., Sato, S., Mimuro, M., Miyashita, H., Tsuchiya, T., et al. (2003) Complete genome structure of Gloeobacter violaceus PCC 7421, a cyanobacterium that lacks thylakoids. *DNA Res* 10, 137–145.

O'Connell, C.M. and Nicks, K.M. (2006) A plasmid-cured Chlamydia muridarum strain displays altered plaque morphology and reduced infectivity in cell culture. *Microbiology* 152, 1601–1607.

Oshima, K., Kakizawa, S., Nishigawa, H., Jung, H.-Y., Wei, W., Suzuki, S., et al. (2004) Reductive evolution suggested from the complete genome sequence of a plant-pathogenic phytoplasma. *Nat Genet* 36, 27–29.

Parkhill, J., Achtman, M., James, K.D., Bentley, S.D., Churcher, C., Klee, S.R., et al. (2000) Complete DNA sequence of a serogroup A strain of Neisseria meningitidis Z2491. *Nature* 404, 502–506.

Rendulic, S., Jagtap, P., Rosinus, A., Eppinger, M., Baar, C., Lanz, C., et al. (2004) A predator unmasked: life cycle of Bdellovibrio bacteriovorus from a genomic perspective. *Science* 303, 689–692.

Shigenobu, S., Watanabe, H., Hattori, M., Sakaki, Y., and Ishikawa, H. (2000) Genome sequence of the endocellular bacterial symbiont of aphids Buchnera sp. APS. *Nature* 407, 81–86.

Ueda, K., Yamashita, A., Ishikawa, J., Shimada, M., Watsuji, T.-O., Morimura, K., et al. (2004) Genome sequence of Symbiobacterium thermophilum, an uncultivable bacterium that depends on microbial commensalism. *Nucleic Acids Res* 32, 4937–4944.

Wu, D., Daugherty, S.C., Van Aken, S.E., Pai, G.H., Watkins, K.L., Khouri, H., et al. (2006) Metabolic complementarity and genomics of the dual bacterial symbiosis of sharpshooters. *PLoS Biol* 4, e188.

Wu, M., Sun, L.V., Vamathevan, J., Riegler, M., Deboy, R., Brownlie, J.C., et al. (2004) Phylogenomics of the reproductive parasite Wolbachia pipientis wMel: a streamlined genome overrun by mobile genetic elements. *PLoS Biol* 2, E69.
